# Development of a systematic approach to conversion of the ileoanal pouch into continent ileostomy

**DOI:** 10.1007/s10151-021-02513-9

**Published:** 2021-08-25

**Authors:** K.-W. Ecker, C. Dinh, N. K. J. Ecker

**Affiliations:** 1 Department of General, Visceral, Vascular, and Pediatric Surgery, University of Saarland, Homburg, Saar Germany; 2 Emeritus Director of the Surgical Department, MediClin Müritz-Klinikum, Fontanestraße 56, 17192 Waren, Germany; 3 Osnabrück, Germany; 4 Ahrensburg, Germany

**Keywords:** Failed IPAA, Restorative proctocolectomy, Salvage operation, Ileal intussusception, Quality of life

## Abstract

Based on practical experience, a systematic approach to conversion of ileal J-pouches into continent ileostomies is developed by defining three types of conversion surgery, each with two subtypes. Type 1 refers to conversion without pouch reconstruction, type 2 to partial pouch reconstruction, and type 3 to complete pouch reconstruction. The subdivisions (a and b) take into account whether the afferent loop of the former pelvic pouch (a) or a higher ileal/jejunal segment of the small intestine (b) is used in conversion and/or reconstruction. The six resulting surgical variants are shown in schematic illustrations with accompanying descriptions of technical details to provide the specialized surgeon with comprehensive technical guidance.

## Introduction

The preferred method of proctocolectomy is ileoanal pouch surgery [ileal pouch–anal anastomosis (IPAA)]. It has replaced its precursor, continent ileostomy (CI), because unlike CI, it preserves the normal defecation route [1]. The common feature of both procedures is the intestinal reservoir. Therefore, IPAA can be converted to CI [2], an operation which was first described in detail by Kusunoki in 1990 [3]. Conversion may become necessary if an IPAA has functionally failed and correction is not possible or has also failed. After careful release from the pelvis, the pouch is usually completed with a nipple valve from the afferent loop [4]. However, this standard conversion does not succeed in all cases, and complete pouch reconstructions often have to be performed [5]. However, this inevitably leads to potentially unnecessary sacrifice of physiologically important small intestine. Two additional technical modifications to avoid this were published by the first author in 1996 [6]. In the meantime, other bowel-sparing techniques have been clinically tested in his practice, so that a systematic approach to conversion surgery can be empirically derived. Herein, these approaches are presented by means of schematic illustrations and detailed descriptions of the corresponding surgical techniques. Our aim is to provide the specialized surgeon with comprehensive technical guidance, rather than reporting surgical results.

## Materials and methods

On the basis of the first criterium (necessity and extent of pouch reconstruction), three main types of conversion are defined:

- **Type 1**: simple conversion (without pouch reconstruction)
- **Type 2**: partial pouch reconstruction required
- **Type 3**: complete reconstruction of a new CI required

According to secondary criteria (site of small intestine removal and need of transposition), two subtypes are defined:

- **a**: Use of the afferent loop
- **b**: Transposition of oral ileum or jejunum

## Results

### Type 1: J-pouch conversion without pouch reconstruction

This is the classic standard conversion. It can always be used in cases where the existing J-pouch is worth preserving and its capacity is sufficient.

- **Type 1a: nipple valve out of afferent loop**

**Fig. 1:**
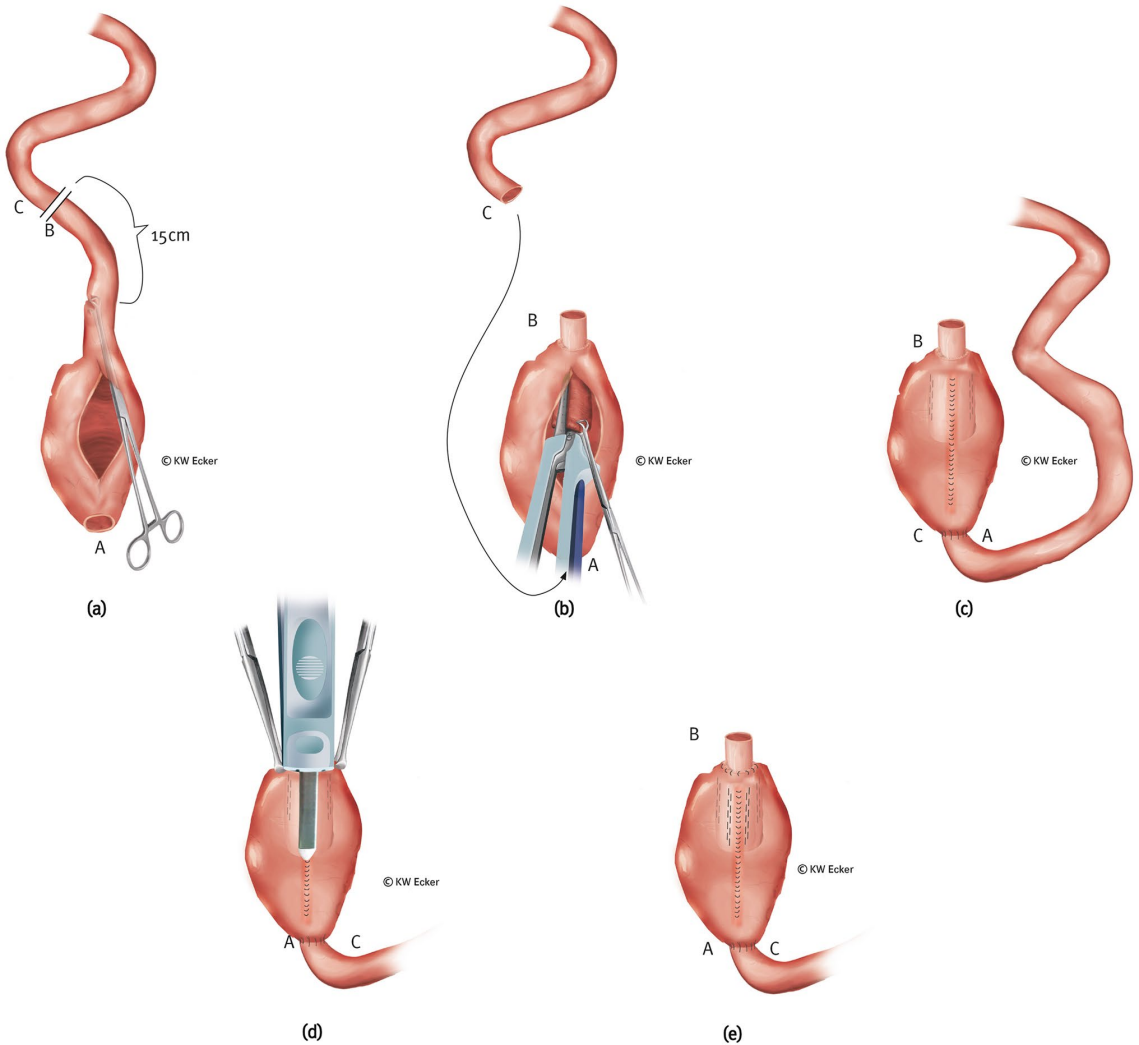
**Conversion without pouch reconstruction using an afferent ileal loop for nipple valve construction**. **a** The former (J-) pouch is detached from the anus above the ileoanal anastomosis and mobilized from the pelvis. Approximately 15 cm orally of the pouch, the afferent loop is divided between B and C. Via a longitudinal pouchotomy (alternatively via the former pouch outlet), the surgeon grasps the former afferent loop with a suitable (e.g., Babcock) clamp to initiate intussusception. **b** After complete intussusception, the nipple valve is formed and stabilized paramesenterically on both sides with two applications of a bladeless linear cutter (= internal valve stabilization). The arrow indicates how the new afferent loop (C) is to be anastomosed with the former pouch outlet (A). **c** The pouchotomy is reclosed by suture and intestinal continuity is restored by anastomosis (C–A). **d** The broader branch of a bladeless linear cutter is inserted as far as possible into the intussusception so that the narrower branch on the outside exactly covers the suture of the pouchotomy. By firing the device, the outer cuff of the nipple valve is connected to the pouch wall with a double clamp suture on each side of the pouch suture (= valve-to-wall fixation). **e** Shown are the fixing rows of staple sutures on both sides of the pouch suture and the inner rows of staple sutures for valve stabilization (translucent). At the pouch shoulder, the outlet canal is connected to the pouch wall with absorbable interrupted sutures (= telescope-securing sutures)

This is the classic standard design of the nipple valve. As the simplest technique, it is suitable in cases where the afferent loop is morphologically intact (Fig. 1).

- **Type 1b: nipple valve out of transposed higher ileum or jejunum**

**Fig. 2:**
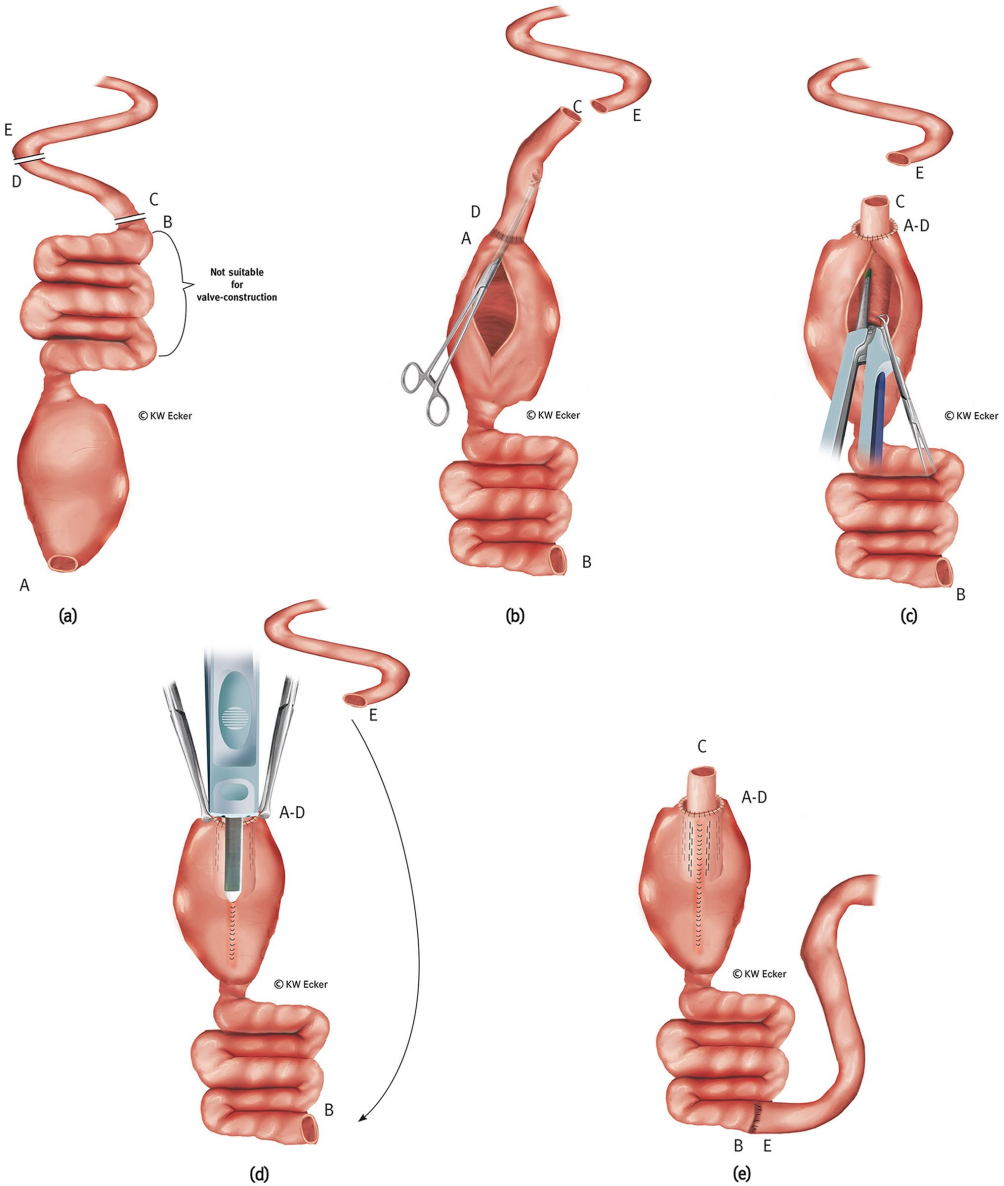
**Conversion without pouch reconstruction using a transposed higher ileal or jejunal segment for nipple valve construction.** **a** The former (J-) pouch is detached from the anus above the ileoanal anastomosis and mobilized from the pelvis. It is morphologically acceptable and therefore reusable. However, although one or more afferent loops are unsuitable for valve formation, they do qualify for passage of intestinal contents and absorption of food components, and are therefore left in continuity with the pouch. From the morphologically intact small intestine cranially, a segment of about 15 cm is isolated between B–C and D–E for valve formation, while maintaining the blood supply. **b** The pouch and preserved afferent loops are rotated by 180°. The isolated small intestinal segment is isoperistaltically anastomosed to the former pouch outlet (A; A–D) by also rotating it by 180°. Via a longitudinal pouchotomy, the transposed small intestinal segment is grasped with a suitable (e.g., Babcock) clamp to initiate intussusception. **c** After intussusception is completed, the nipple valve is formed and stabilized paramesenterically on both sides by staple applications with a bladeless linear cutter (= internal valve stabilization). **d** The pouchotomy is closed by continuous suture. The broader branch of a bladeless linear cutter is inserted as far as possible into the intussusception so that the narrower branch on the outside exactly covers the pouchotomy suture. By firing the device, the outer cuff of the nipple valve is connected to the pouch wall with a double clamp suture on each side of the pouch suture (= valve-to-wall fixation). The arrow indicates how the new afferent loop (E) is anastomosed with the former afferent loop (B). **e** Shown are the fixing rows of staples on both sides of the pouch suture and the inner rows of staples for valve stabilization (translucent). At the pouch shoulder, the outlet canal is connected to the pouch wall with absorbable interrupted sutures (= telescope-securing sutures). Intestinal continuity is restored (B–E)

In cases where the lumen of the afferent loop is too narrow (precluding intussusception) or too wide (risk of floppy valve), and/or in case of scarring or inflammatory or fibrodesmoid changes, it is advisable to move to a higher segment of the small intestine and transpose it. In this case, the afferent loop can remain in continuity if there is no clinically relevant obstruction of the passage prior to surgery (Fig. 2).

### Type 2: J-pouch conversion with partial pouch reconstruction

There are two situations in which the J-pouch should be reconstructed or augmented. First, in cases of significant adhesions in the small pelvis, one of the two limbs of the J-pouch may be so injured during mobilization that it must be resected. These are usually patients in whom the indication for conversion results from the sequelae of septic complications following IPAA surgery. On the other hand, there are cases in which, after injury-free mobilization, the pouch capacity proves insufficient. These are often conversion candidates who were plagued by excessive evacuation frequency after IPAA surgery that could not be influenced by any measure.

- **Type 2a: nipple valve plus pouch augmentation out of the afferent loop**

**Fig. 3:**
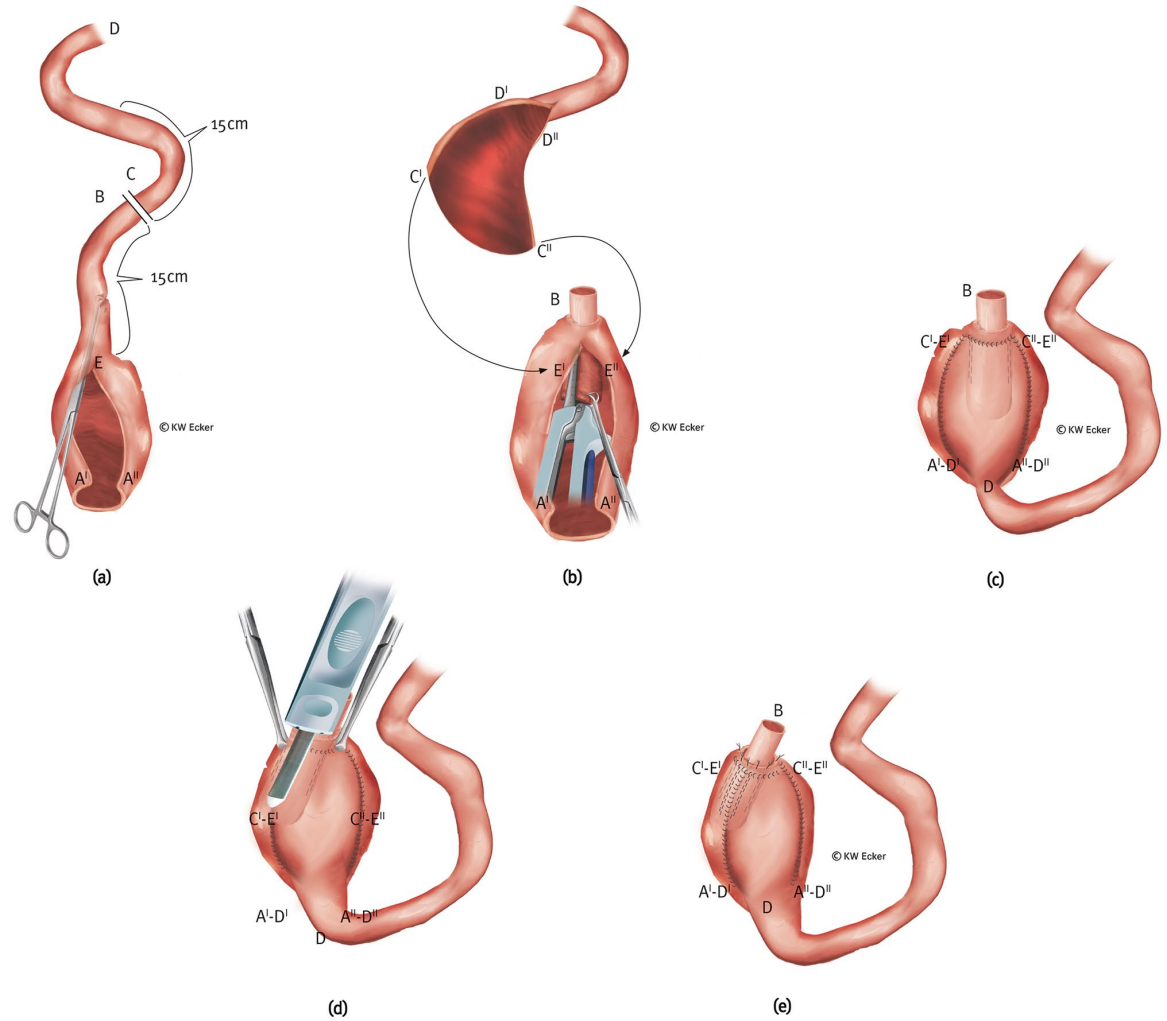
**Conversion with partial pouch reconstruction using an afferent ileal loop for both pouch reconstruction and nipple valve construction**. **a** The former (J-) pouch is detached from the anus above the ileoanal anastomosis, mobilized from the pelvis, and incised lengthwise. Approximately 15 cm orally of the pouch, the afferent loop is transected between B and C. Over the opened pouch, a suitable (e.g., Babcock) clamp is used to grasp the former afferent loop to initiate intussusception. **b** The nipple valve is formed by complete intussusception and stabilized paramesenterically on both sides with two applications of a bladeless linear cutter (= internal valve stabilization).The arrows indicate how the new afferent loop is anastomosed with the longitudinally opened pouch after an appropriate contramesenteric longitudinal incision, whereby C' is positioned adjacent to E' and C'' adjacent to E'', in order to then connect D' to A' and D'' to A''. **c** All anastomotic sutures are completed continuously, thus restoring intestinal continuity. **d** The broader branch of a bladeless linear cutter is inserted as far as possible into the nipple valve intussusception so that the narrower branch externally covers one of the two longitudinal suture lines exactly. By firing the device, the outer cuff of the nipple valve is connected to the pouch wall with a double row of clamps on each side of the selected pouch suture (= valve-to-wall fixation). **e** Shown are the fixing staple rows on both sides of the pouch suture and the inner staple rows for valve stabilization (translucent). At the pouch shoulder, the outlet canal is connected to the pouch wall with absorbable single button sutures (= telescope-securing sutures)

This is the standard reconstruction/augmentation in cases where the afferent loop is suitable for nipple valve construction (Fig. 3).

- **Type 2b: nipple valve plus pouch augmentation out of a transposed higher ileal or jejunal segment**

**Fig. 4:**
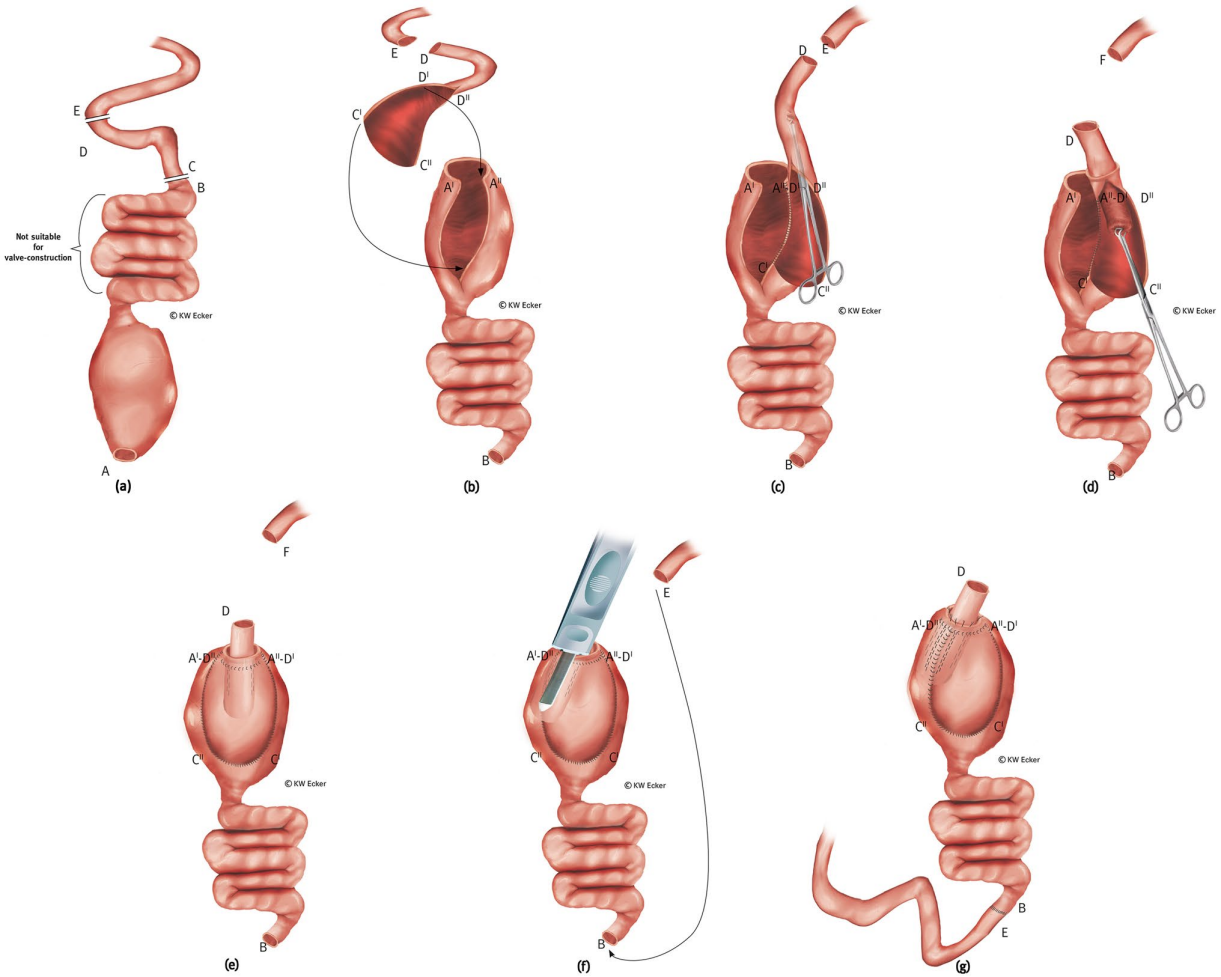
**Conversion with partial pouch reconstruction using a higher ileal or jejunal segment for both pouch reconstruction and nipple valve construction.** **a** The former (J-) pouch is detached from the anus above the ileoanal anastomosis and mobilized from the pelvis. Although morphologically intact, it appears too small or was damaged during preparation. In principle, the existing pouch is worth preserving. Although one or more afferent loops are unsuitable for valve formation, they do qualify for passage of intestinal contents and absorption of food components. They are therefore left in continuity with the residual pouch. From the oral morphologically intact small intestine, a segment of about 15 cm is isolated between B–C and D–E for valve formation, while maintaining the blood supply. **b** After the too small pouch has been incised lengthwise or the margins of the damaged pouch have been adjusted, the pouch is rotated by 180°. The small intestinal segment designated for pouch reconstruction (augmentation) is (also) incised contramesenterically in the corresponding length. The arrows between D' and A' and D'' and A'' indicate how augmentation is performed. **c** After completing one of the two longitudinal sutures, a clamp is inserted into the lumen of the designated small intestinal segment to initiate intussusception of the nipple valve. **d** After complete intussusception, the nipple valve is formed and stabilized paramesenterically on both sides via two applications with a bladeless linear cutter (= internal valve stabilization). **e** All anastomoses are completed by continuous sutures to fully close the pouch. The clamps for stabilizing the nipple valve are shown translucently. **f** The broader branch of a bladeless linear cutter is inserted as far as possible into the intussusception of the nipple valve so that the narrower branch externally covers one of the two longitudinal sutures exactly. By firing the device, the outer cuff of the nipple valve is connected to the pouch wall with a double row of clamps on both sides of the selected pouch suture (= valve-to-wall fixation). **g** Shown are the fixing rows of staple sutures on both sides of the pouch suture and the inner rows of staples (translucent). At the pouch shoulder, the outlet canal is connected to the pouch wall with absorbable interrupted sutures (= telescope-securing sutures). Bowel continuity is completed by enteroanastomosis between B and E

The indication for or field of application of this variant corresponds to the conditions described for type 1b (Fig. 4).

### Type 3: S-pouch construction as complete pouch reconstruction

In rare cases (preparatory destruction, circumscribed severe pouchitis, and scarring, inflammatory, or fibrodesmoid shrinkage), the J-pouch is non-salvageable. If no contraindications for the construction of a second pouch are present, complete new construction of the CI can be performed.

- **Type 3a: new CI out of neoterminal ileum**

**Fig. 5:**
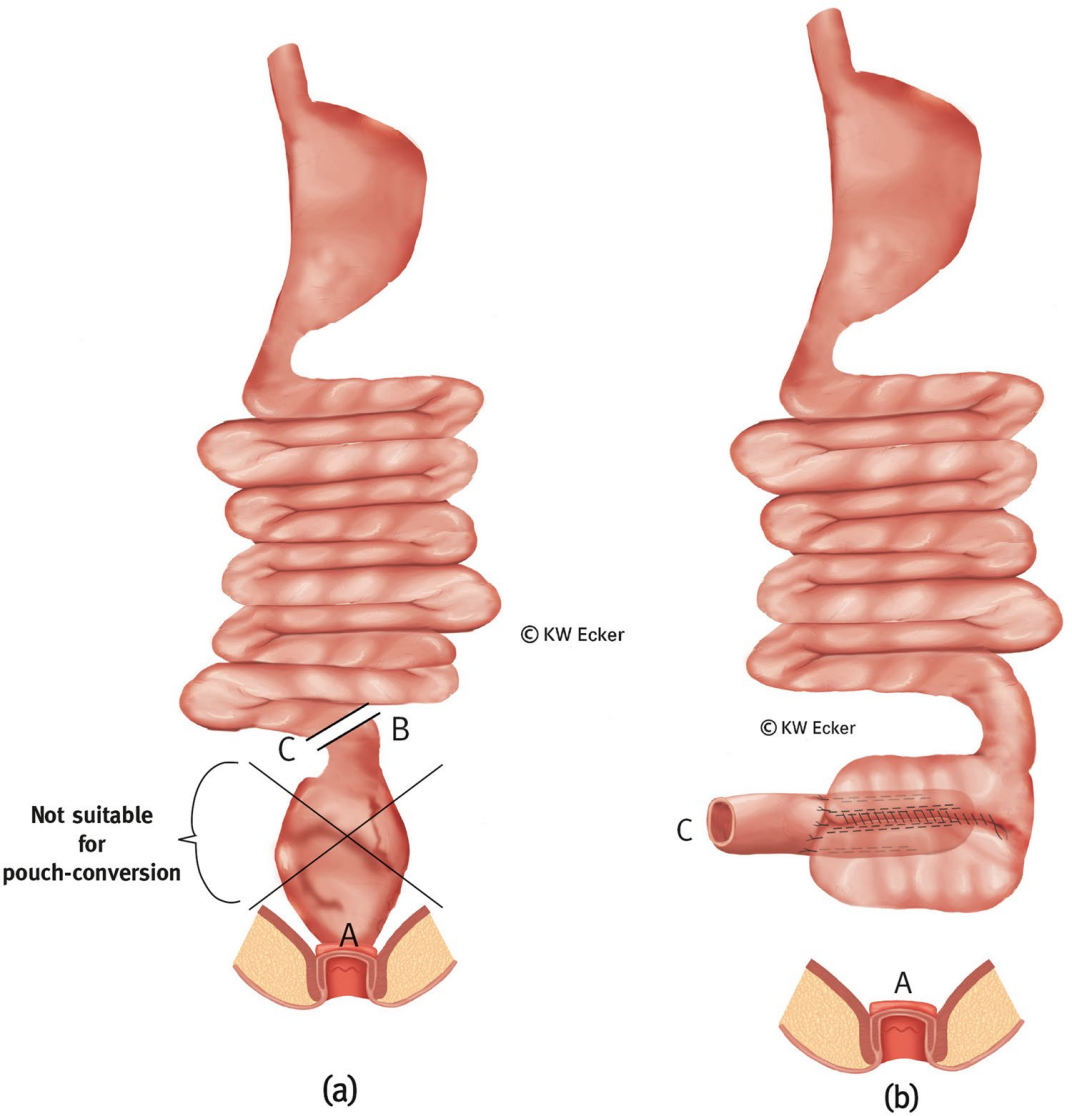
**Conversion by complete reconstruction of a new S-pouch using neoterminal ileum.** **a** The pouch unsuitable for transformation is resected and preserved for histologic examination. This may be accompanied by intersphincteric proctectomy or the sphincter is closed at the upper margin with staples or hand-suture (A). Starting at the neoterminal ileum (C), the reconstruction of a continent ileostomy (CI) is prepared. **b** The reconstruction of a CI (S-design) is finished and ready for implantation in the right lower abdomen. The residual sphincter is closed with suture (A)

This is the standard new construction of CI in cases where the afferent loop is morphologically suitable (Fig. 5).

- **Type 3b: new CI out of transposed higher ileum or jejunum**

**Fig. 6:**
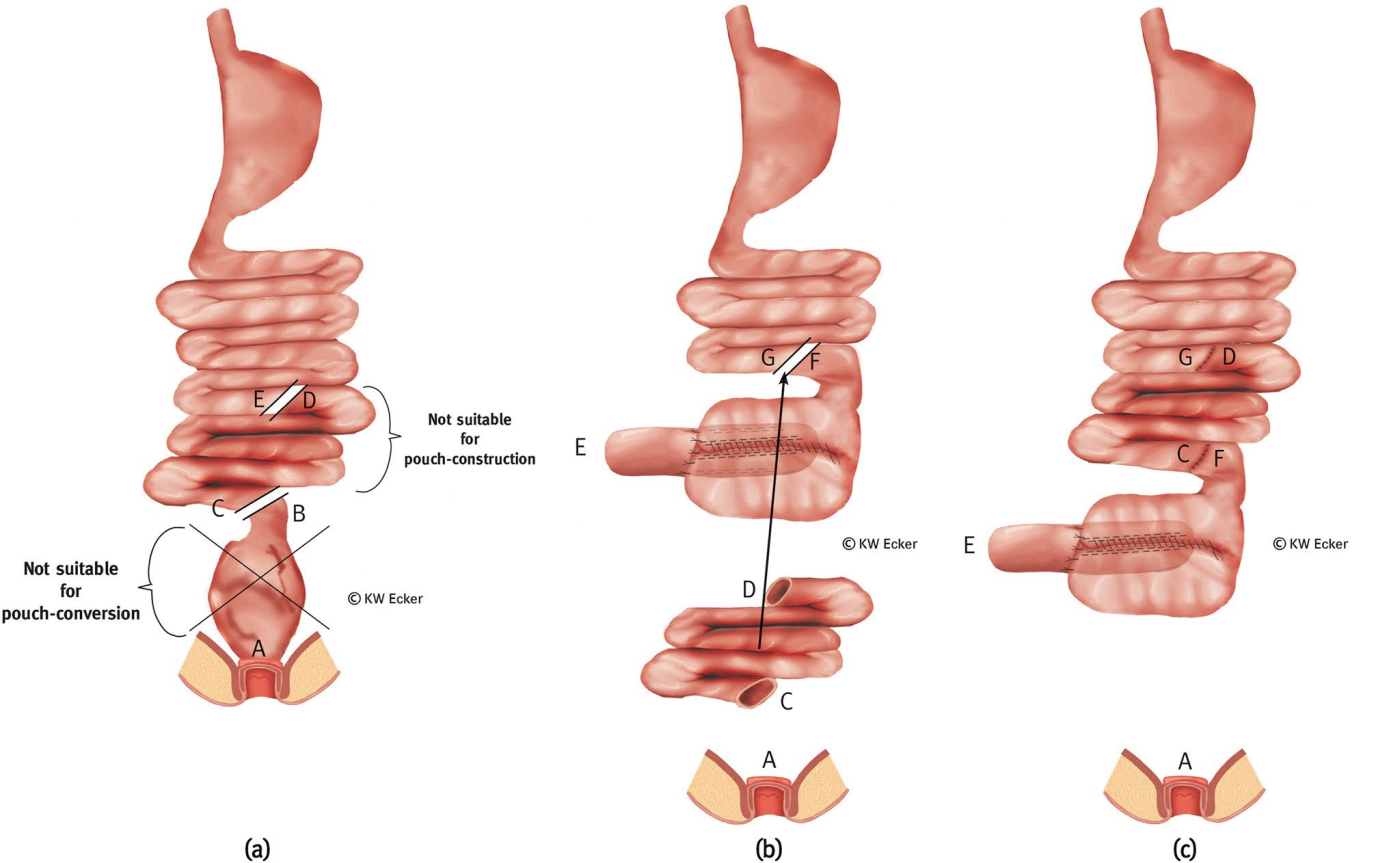
**Conversion by complete reconstruction of a new S-pouch using a higher ileal or jejunal segment transposed to the end of the small intestine**. **a** The pouch unsuitable for conversion is resected between B and C and preserved for histologic examination. This may be accompanied by intersphincteric proctectomy or the sphincter is closed at the upper edge with staples or hand-suture (A). Although one or more afferent loops are unsuitable for valve formation, they are suitable for passage of intestinal contents and absorption of food components. They are therefore reserved for later transposition while maintaining the blood supply. The small intestine is cut orally between D and E. Starting from E, the new continent ileostomy (CI) is constructed. **b** The new CI is completed and the afferent loop is cut between E and G. The reserved segment of small intestine (C, D) is transposed as indicated by the arrow. **c** Transposition of the small intestinal segment is completed by restoration of intestinal continuity (C–F and B–G). The new CI is ready for implantation in the right lower abdomen. The remaining sphincter is closed with suture (A)

This is a reserve method for reconstruction of the CI. It can be used in cases with afferent loop conditions, as described in 1b. This variant is particularly suitable in Crohn's disease (CD), since the jejunum is often spared from recurrences of inflammation in this underlying disease for the rest of the patient's life [7] (Fig. 6).

## Discussion

We focus on the various modifications of an interesting surgical procedure and the rationale behind it, not on the clinical results. We successfully performed a total of 26 surgeries using all six surgical variants between 1988 and 2015. Publication of the clinical results is underway. The emphasis herein is thus on the technical details. We assume that the corresponding schematic illustrations are largely self-explanatory for the specialized surgeon. Nevertheless, surgical implementation of salvage operations is always challenging and sometimes difficult [8]. Interested surgeons are advised to attend experienced centers and perform their first operations under the supervision of a specialized pouch surgeon. In doing so, they will learn that there is almost always a realizable solution. Thus, in some cases of type 2 conversions, it may be useful to combine technical components from type 2a with those from type 2b. This means, for example, valve formation from the afferent loop, but pouch reconstruction from a higher small bowel segment or vice versa. However, one must first get a "feel" for the techniques under supervision.

In selected cases, CI is worth considering and offers the advantage of complete control over fecal evacuation, with a much better quality of life (QoL) compared to conventional ileostomy [2, 6]. Consequently, conversion of IPAA to CI has two goals. The first is to improve QoL by establishing artificial fecal continence [3–5]. The second goal is concerned with preserving normal physiology as far as possible [6, 9]. Usually, a failed pouch is excised or excluded. This means that in the case of an end or loop ileostomy, 50–75 cm of small bowel is no longer available for resorption. It has long been known that clinically meaningful metabolic disturbances may result, including severe syndromes of loss of water and electrolytes, as well as renal failure and psychological disorders [10, 11]. Therefore, the existing pouch should be reused whenever feasible.

From a technical point of view, first and foremost, the pouch should be dissected from the pelvis as atraumatically as possible. Minor damage can be repaired while preserving the pouch [11]. However, it is not uncommon for one of the loops of the J-pouch to be non-salvageable, either in part or totally. This is why it must be replaced if the pouch is not to be sacrificed completely. In addition, there are patients in whom the afferent loop is suitable for neither valve formation nor pouch reconstruction for various reasons (scars, thickening, fibrodesmoid changes, etc.). In these situations, if no passage disorders were present preoperatively, it can be assumed that they will not occur postoperatively if these bowel segments remain in continuity. Single or multiple intestinal transpositions within the abdomen may help to avoid unnecessary losses. Thus, complete reconstructions are necessary in desperate situations only. Transposition of jejunum, as previously described by Barnett, may represent a further but important “trick,” not only in CD [7].

These approaches, categorized into three primary types, each with two secondary subtypes, take into account all situations occurring in clinical practice. Moreover, it has been shown that categorizing the approaches is very helpful from a didactic perspective. Critics may argue that this is a sophistical over-differentiation, and that the complexity of the described variants could lead to increased complications.

## Conclusions

The systematic derivation of technical variants of conversion surgery expands the surgical armamentarium of the specialized surgeon to all underlying conditions encountered in practice. Thus, it is at least theoretically possible to save more patients with failed IPAA from the fate of a conventional ileostomy. Practical feasibility requires further evaluation in larger controlled studies.

## Data Availability

The data and all material used is secured digitally by the corresponding author.

## Code availability

No code available.

## References

[1] Fazio V, Kiran R, Remzi F (2013). Ileal pouch anal anastomosis anastomosis : analysis of outcome and quality of life in 3707 patients. Ann Surg.

[2] Hultén L (1985). The continent ileostomy (Kock’s pouch) versus the restorative proctocolectomy (pelvic pouch). World J Surg.

[3] Kusunoki M, Sakanoune Y, Shoji Y (1990). Conversion of malfunctioning J pouch to Kock’s pouch. Case report. Acta Chir Scand.

[4] Hultén L, Fasth S, Hallgren T, Öresland T (1992). The failing pelvic pouch conversion to continent ileostomy. Int J Colorectal Dis.

[5] Behrens DT, Paris M, Luttrell JN (1999). Conversion of failed ileal pouch-anal anastomosis to continent ileostomy. Dis Colon Rectum.

[6] Ecker KW, Haberer M, Feifel G (1996). Conversion of the failing ileoanal pouch to reservoir-ileostomy rather than to ileostomy alone. Dis Colon Rectum.

[7] Barnett WO (1986). The continent jejunal reservoir in Crohn’s colitis. J Miss State Med Assoc.

[8] Wasmuth HH, Myrvold HE (2009). Durability of Ileal pouch–anal anastomosis and continent ileostomy. Dis Colon Rectum.

[9] Börjesson L, Oresland T, Hulten L (2004). The failed pelvic pouch: conversion to a continent ileostomy. Tech Coloproctol.

[10] Delin K, Fasth S, Andersson H (1984). Factors regulating sodium balance in proctocolectomized patients with various ileal resections. Scand J Gastroenterol.

[11] Bengtson J, Solberg A (2019). The Kock pouch.

